# Moral Reminders Do Not Reduce Symptom Over-Reporting Tendencies

**DOI:** 10.1007/s12207-017-9303-9

**Published:** 2017-11-11

**Authors:** Isabella J. M. Niesten, Wenke Müller, Harald Merckelbach, Brechje Dandachi-FitzGerald, Marko Jelicic

**Affiliations:** 1 Clinical Psychological Science, Maastricht University, P.O. Box 616, 6200 MD Maastricht, The Netherlands; 2 Department of Psychiatry and Psychology, School for Mental Health and Neuroscience, Maastricht University Medical Centre, Maastricht, The Netherlands

**Keywords:** Symptom over-reporting, Feigning, Cognitive dissonance, Moral primes, Moral licensing/cleansing, Self-serving justifications

## Abstract

Is presenting patients with moral reminders prior to psychological testing a fruitful deterrence strategy for symptom over-reporting? We addressed this question in three ways. In study 1, we presented individuals seeking treatment for ADHD complaints (*n* = 24) with moral primes using the Mother Teresa Questionnaire and compared their scores on an index of symptom over-reporting (i.e., the Structured Inventory of Malingered Symptomatology, SIMS) with those of unprimed patient controls (*n* = 27). Moral primes slightly decreased SIMS scores, but the effect was not significant. In study 2, we took a different approach to activate moral categories: we recruited individuals seeking treatment for ADHD complaints and asked some of them to sign a moral contract (i.e., prime; *n* = 19) declaring that they would complete the tests in an honest way and compared their scores on the SIMS and standard clinical scales measuring self-reported psychopathology with those of unprimed patient controls (*n* = 17). Again, we found no convincing evidence that moral cues suppress symptom over-reporting. In study 3, we gave individuals from the general population (*N* = 132) positive, negative, or neutral moral primes and implicitly induced them to feign symptoms, after which they completed a brief validated version of the SIMS and an adapted version of the b Test (i.e., an underperformance measure). Again, primes did not affect over-reporting tendencies. Taken together, our findings illustrate that moral reminders are not going to be useful in clinical practice. Rather, they point towards the importance of studying contextual and individual difference factors that guide moral decision-making in patients and may be modified to discourage symptom over-reporting.

For a long time, experts’ attitude towards intentional symptom over-reporting (for example, feigning, and malingering) was dominated by blissful ignorance. Most professionals assumed that the phenomenon was rare and confined to forensic cases and, as a result, they were reluctant to consider its presence. The past two decades have witnessed a radical change in perspective. With the introduction of symptom validity tests (SVTs; see for an overview Young, 2014), it became clear that patients across various settings might exaggerate their symptoms (Alwes, Clark, Berry, & Granacher, 2008; Ardolf, Denney, & Houston, 2007; Dandachi-FitzGerald, Ponds, Peters, & Merckelbach, 2011). For example, Dandachi-FitzGerald et al. (2011) found that 30% of their non-forensic psychiatric outpatients obtained symptom profiles indicative of symptom over-reporting. Studies have shown that ignoring symptom exaggeration does not only have dire consequences in terms of societal costs (e.g., healthcare and work absenteeism costs; e.g., Chafetz & Underhill, 2013) but can also result in a biased understanding of the etiology of true psychopathology (Merckelbach, Langeland, de Vries, & Draijer, 2014; Rienstra et al., 2013; Rohling et al., 2011). Thus, Merckelbach et al. (2014) observed a typical dose-response relationship between abuse severity and later psychopathology among non-exaggerating sexual abuse victims but not among those who exaggerated their symptoms. Likewise, Rienstra et al. (2013) found the usual brain-behavior correlation between hippocampal volume and memory performance in patients with mild cognitive complaints but not in the subgroup of patients showing non-credible performance on an SVT. Clearly, such findings call for strategies to discourage symptom over-reporting tendencies. But what should such strategies look like?

Past efforts have either focused on providing explicit warnings prior to test administration (e.g., referring to SVTs in the test battery; Etherton & Axelrod, 2013; Gorny & Merten, 2006; King & Sullivan, 2009; Schenk & Sullivan, 2010; Sullivan & Richer, 2002) or corrective feedback after SVT failure (Merckelbach, Dandachi-FitzGerald, van Mulken, Ponds, & Niesten, 2015; Suchy, Chelune, Franchow, & Thorgusen, 2012; Carone, 2017). Unfortunately, both strategies have their limitations. Some authors have, for instance, argued that providing warnings may not be a good idea as this approach might, in fact, promote more sophisticated feigning in certain cases (e.g., Youngjohn, Lees-Haley, & Binder, 1999). In line with this, practice recommendations advice clinicians to make examinees aware that effort and honesty are essential during testing but warn against explicit mention of SVTs as this could undermine the validity of collected data (e.g., Bush, Heilbronner, & Ruff, 2014; Iverson, 2006). Providing patients who engage in over-reporting with corrective feedback is also not without problems. Suchy et al. (2012) noted that providing feedback does not have any effect in a sizeable minority (33%) of patients. Furthermore, although post hoc feedback seemed to decrease symptom exaggeration on subsequent testing in the majority of patients, the authors found that symptom scores obtained by these individuals rarely normalize to the extent that they match those of non-feigning individuals (see also Merckelbach et al., 2015). What further limits the current body of research on reduction strategies is that it is seldom inspired by theoretical frameworks that explain their effects on symptom over-reporting.

Why do current reduction strategies only have a modest effect at best? One explanation can be found in cognitive dissonance theory (see also Merckelbach et al., 2015): Because people prefer to see themselves as moral beings, acting inconsistently with this self-definition causes an aversive state of arousal or dissonance (for examples see Aquino & Reed, 2002; Aronson, 1992; Cooper, 2007; Stone & Cooper, 2001; Merckelbach & Merten, 2012; Niesten, Nentjes, Merckelbach, & Bernstein, 2015; Niesten, Merckelbach, van Impelen, Jelicic, Manderson, & Cheng, 2017). This uncomfortable state drives individuals to protect their self-concept, oftentimes by coping with inconsistencies via a defensive distortion of information. To this end, they may adopt self-serving justifications, biases, and other forms of denial. In the case of symptom over-reporting, dissonance is likely to arise due to a salient conflict between internal standards (i.e., “I am an honest and healthy individual”) and the knowledge that one’s symptoms are, in reality, not as severe as reported (i.e., “I am being dishonest”). Studies support the idea that the act of feigning is dissonance-inducing to some individuals (e.g., Niesten et al., 2015; Niesten et al., 2017). Furthermore, both clinical cases and empirical data suggest that people may resolve this dissonance through a self-deceptive reevaluation of initially feigned symptoms as signs of genuine illness (i.e., “I really do suffer from symptom X”; Kunst, Aarts, Frolijk, & Poelwijk, 2015; Merckelbach, Jelicic, & Pieters, 2011; for an extensive theoretical analysis of dissonance in the context of symptom over-reporting see Bayer, 1985). This way, feigned symptoms may over time evolve into a less conscious, yet potentially chronic, form of symptom production. Importantly, both explicit warnings and feedback may activate dissonance-related emotions—e.g., related to prior acts of feigning—that threaten the moral self-concept and foster (further) internalization of over-reported symptoms. To illustrate, Merckelbach et al. (2015) provided undergraduates with a legal case vignette and the option to over-report symptoms on an SVT. After test completion, participants were confronted with their SVT failure and asked to complete a symptom list. Those who had been confronted reported significant feelings of guilt (i.e., dissonance) and showed elevated symptom scores when compared with controls. This suggests that cognitive dissonance might account for the observation that overall, warnings and feedback have little corrective potential.

That symptom over-reporting may induce dissonance and foster residual symptoms simultaneously suggests there might be a more effective strategy to reduce over-reporting tendencies. That is, if people experience dissonance *after* they engaged in symptom over-reporting, making moral self-standards salient *before* the actual act may deter over-reporting. Indeed, alongside a vast amount of research illustrating how post-decisional dissonance can lead individuals to justify their—sometimes undesirable—actions (see for an overview Cooper, 2007), there is now a large corpus of literature showing that interventions that apply pre-decisional or *anticipated* dissonance can positively affect subsequent behavior. Researchers have theorized that when dissonance is aroused prior to the possibility to execute behavior, it helps individuals become aware of their own moral strivings and increases their commitment to act in a self-consistent fashion. In their review on dissonance-based interventions, Freijy and Kothe (2013) concluded that anticipated dissonance reduces various undesirable behaviors, including sexual risk behavior, smoking, alcohol use, and reckless driving (see also Stone & Fernandez, 2008). Likewise, numerous examples within social psychology, behavioral economics, and behavioral ethics demonstrate that activating moral standards prior to violations can curb deception tendencies in domains that show conceptual overlap with deliberate symptom over-reporting. Germane to this is a study by Mazar, Amir, and Ariely (2008), who examined whether the activation of moral standards decreases cheating. Prior to test completion, students either cited the Ten Commandments or recalled ten books they had read in high school (i.e., controls). The former group was found to cheat significantly less than the latter, which made the authors conclude that an intervention as unobtrusive as a moral reminder may discourage unethical behavior (see for similar findings; Randolph-Seng & Nielsen, 2007). The successes that neighboring fields dealing with dishonest responding have achieved with moral reminders suggest that exploring their potential in the context of symptom over-reporting is a legitimate endeavor.

Recently, our research group reported an initial attempt to activate moral standards to discourage over-reporting in outpatients seeking treatment for attention deficit hyperactivity disorder (ADHD). For this pilot study, Merckelbach and Collaris (2012) developed the Mother Teresa Questionnaire (MTQ), a list of ten statements intended to prime moral standards. Although no significant difference in symptom scores (including those on an SVT) emerged between patients who had been primed with the moral questionnaire (*n* = 10) and controls (*n* = 10), the authors did observe a trend in the hypothesized direction (*p* = .11). In a more recent study, Horner, Turner, van Kirk, and Denning (2017) asked patients to sign a handout that stressed the importance of honest responding and compared their scores on an SVT with those of patients who had been given a neutral handout. While they did not observe meaningful differences across conditions, they did obtain a lower frequency of SVT failures among patients with a self-reported interest in disability benefits. This led the authors to recommend their intervention as a promising, cost-free method for reducing the occurrence of invalid data.

With this research in mind, the present paper aimed to further test the idea that moral reminders suppress over-reporting tendencies. Thus, in study 1, we tested the effect of the Mother Teresa prime in an additional number of patients to see whether any priming effect may have been obscured by an underpowered sample size in the original pilot study (i.e., Merckelbach & Collaris, 2012). In study 2, we set out to boost the effect of our moral reminder by asking patients to sign a moral contract. The samples in these studies consisted of outpatients referred for ADHD complaints. We were not specifically interested in genuine and feigned ADHD but rather selected this category of patients because a diagnosis of ADHD can provide individuals with several benefits, including psychostimulant medication and academic advantages. Accordingly, symptom over-reporting is not uncommon in this group, with base rate estimates approaching 30% and occasionally even 45% (Sullivan, May, & Galbally, 2007), although base rates in the order of 20% have also been reported (e.g., Clemow & Walker, 2014; Marshall et al., 2010; Suhr, Hammers, Dobbins-Buckland, Zimak, & Hughes, 2008). Based on cognitive dissonance theory, we expected patients who had been presented with a moral reminder to anticipate dissonance and, as a consequence, show *less* symptom over-reporting than their non-primed counterparts.

In study 3, we took a different approach and employed an induced-feigning paradigm in participants recruited from the general population to more closely examine whether moral reminders have the power to reduce symptom over-reporting tendencies. The practical relevance is obvious: If effective, moral reminders may provide clinicians with a novel, non-invasive, and theoretically well-grounded method to reduce over-reporting and its societal costs (see also Horner et al., 2017).

## Study 1: Moral Primes

### Method

#### Participants

Participants were recruited at PsyQ, an outpatient mental health clinic located in Maastricht, the Netherlands. Similar to Merckelbach and Collaris (2012), participants had been referred to the clinic for a possible diagnosis of ADHD. In total, 60 individuals—including the participants already tested by Merckelbach and Collaris (2012; *n* = 20)—participated in the study. These individuals were randomly allocated to the prime (*n* = 29) or no-prime condition (*n* = 31) following a coin toss procedure. Nine participants had missing values on our outcome measure and were therefore excluded from the analyses. The final sample consisted of 51 participants (32 men; *M*_age_ = 32.3 years, SD = 10.4), of whom 24 were presented with the prime and 27 were not. Both the standing ethical committee of the Faculty of Psychology and Neuroscience of Maastricht University and the clinic’s local committee approved the study.

#### Measures and Procedure

Participants received verbal and written information regarding the study and were asked to give their consent. Importantly, this information did not make explicit reference to SVTs nor did it give an indication regarding our hypotheses or conditions. Subsequently, as part of a routine neuropsychological evaluation, they completed the Structured Inventory for Malingered Symptomatology (SIMS; Cronbach’s alpha = .94; Smith & Burger, 1997; van Impelen, Merckelbach, Jelicic, & Merten, 2014). The SIMS is a 75-item symptom validity instrument that screens for symptom exaggeration across several symptom domains, including amnesia, neurological impairment, psychosis, affective disorders, and (low) intelligence. Each domain is assessed by means of 15 yes/no items. “Yes” items, as well as some “No” items (i.e., after recoding), can be summed into a total score (range 0–75). Based on previous research, scores above the cut off of 16 should raise suspicion about feigning (van Impelen et al., 2014).

Whereas participants in the no-prime condition (i.e., controls) completed the SIMS directly after giving consent, participants in the prime condition were first exposed to moral primes using the MTQ (Merckelbach & Collaris, 2012) and then completed the SIMS. Briefly, the MTQ consists of ten questions that tap into ethical issues as to trigger individuals’ awareness of moral norms (e.g., “If I would have to choose between a nice evening out with a friend or a visit to a lonely and ill family member, I would choose to visit the family member”; see Appendix A for other items). All questions are answered in a “Yes,” “I don’t know,” or “No” format.^1^ Importantly, the MTQ is not intended as a measure but rather as a prime to activate people’s awareness of moral standards. After completing all measures, participants received a debrief form stating that the study intended to improve the accuracy of neuropsychological test results.

### Results and Discussion

Table 1 displays SIMS mean scores per condition.^2^ Although participants in the prime condition obtained somewhat lower SIMS scores than those in the no-prime condition, an independent *t* test revealed that this difference was not statistically significant (*t* (49) = .73, *p* > .05, Cohen’s *d* = −.20). Using the recommended cutoff of 16, 9 out of 27 (33%) in the no-prime condition versus 7 out of 24 (29%) in the prime condition exhibited raised levels of over-reporting. This difference was not significant (*χ* ^2^ (1) = .10, *p* > .05, two-tailed). Next, we carried out *t* tests to compare the two conditions with regard to their scores on the subscales of the SIMS (see Table 1). Again, no differences reached significance, with all *t*s < 1 and all *p*s > .01 (i.e., after Bonferroni correction: *α* = .05/five subscales).

**Table 1:** Study 1: mean scores (SD) on the SIMS and SIMS subscales per condition

	Condition	
	Prime (*n* = 24)	No prime (*n* = 27)
Total SIMS	12.4 (10.5)	14.7 (12.0)
NI scale	2.3 (3.0)	2.3 (3.0)
AF scale	4.6 (3.9)	5.4 (2.7)
P scale	0.9 (1.3)	1.6 (2.9)
LI scale	1.3 (1.5)	1.4 (2.8)
AM scale	3.3 (3.5)	4.1 (3.0)

*SIMS* Structured Inventory of Malingered Symptomatology, *NI* neurological impairment, *AF* affective disorders, *P* psychosis, *LI* low intelligence, *AM* amnestic disorders

As another approach to our data, we calculated the Bayesian factor with version 0.9.8 of Bayes Factor Package software (see Morey & Rouder, 2011). The Bayesian factor gives a numerical estimate of the extent to which the data fit better with the alternative hypothesis—priming suppresses symptom exaggeration—than the null hypothesis. For SIMS total scores, we found a Bayesian factor of 1.22, which is in favor of the null rather than the alternative hypothesis.

Finally, we took the effectiveness of our moral manipulation into account. The Pearson correlation between MTQ and SIMS scores was not significant (*r* = −.10, *p* > .05). Participants presented with the MTQ obtained a mean score of 7.8 out of 10 (SD = 1.6, range = 5–10). We selected only those participants exceeding an arbitrary cutoff of ≥ 8 (out of 10 items) and compared their SIMS scores (*n* = 15) with those of controls (*n* = 27). Although these participants (*M* = 10.4, SD = 8.7) scored lower on the SIMS than controls (*M* = 14.7, SD = 12.0), this difference did not reach significance (*t* (40) = 1.23, *p* > .05, Cohen’s *d* = −.39). These findings suggest that the Mother Teresa prime was not strong enough to activate moral categories. Thus, testing an additional number of participants with the Mother Teresa prime did not reveal a significant effect of moral reminders on SIMS scores, and the borderline significant effect reported by Merckelbach and Collaris (2012) did not emerge with this larger sample.

In retrospect, our use of the MTQ prime as a tool to reduce over-reporting may have been naïve: a growing body of research suggests that people do not always act upon a need for consistency but sometimes rely on a balancing system that regulates moral self-concept by analyzing evidence of previous moral and immoral acts. Via this mechanism, moral reminders can be interpreted as an affirmation of a positive moral self rather than as a motivator to exhibit consistent behavior (e.g., Steele & Liu, 1983). In the worst case, this activates moral licensing; when individuals believe there is more evidence of their morality (i.e., credits) than immorality (i.e., debits), they are less susceptible to dissonance and may feel more entitled to opt for unethical choices (Effron & Conway, 2015; Monin & Miller, 2001). As a demonstration, Jordan, Mullen, and Murnighan (2011) found that participants recalling past moral behaviors cheated more than those recalling past immoral behaviors (see also Cascio & Plant, 2015). One could argue that to decide whether or not the MTQ statements applied to them, our participants were required to recall and weigh their past behaviors. Given that statements in the MTQ are framed in such a manner that the moral choice is the default, this may have not required restoring of the moral self-concept. Rather, it may have stimulated a positivity bias and, potentially, given some patients the leeway to license over-reporting (i.e., “I am generally an honest person, so exaggerating my symptoms is not that bad”). Thus, moral reminders may, at times, have no impact and induce a backfire effect if they signal to individuals that they are (already) of good moral character but do not involve them in an active pursuit of that goal. Therefore, moral reminders might better be framed in such a manner that they do not activate recollections of past desirable behavior but stimulate a focus on ethical considerations in the here and now instead. Indeed, moral priming may have worked in the studies by, for example, Mazar et al. (2008) simply due to the fact that the Ten Commandments pertain to ongoing commitment to moral values and refrain from focusing on the past.

Several authors have also suggested that for moral reminders to be effective, they are best accompanied by an element of self-awareness. Awareness of oneself while being in an ethically tempting situation has been proposed to automatically activate a comparison of the self against standards, making discrepancies between conflicting goals (e.g., the desire to obtain benefits through over-reporting and the internal desire to be a moral individual) more salient and thus more dissonance-inducing (Shu, Mazar, Gino, Ariely, & Bazerman, 2011). If self-awareness is increased *prior* to the opportunity to behave unethically, this motivates people to be honest as this helps them to maintain a positive self-concept (Cooper, 2007; Shu et al., 2011). In support of this theory, and across various experiments as well as in naturalistic settings, Shu et al. (2011) tested cheating behavior when participants signed their name prior or after an opportunity to cheat. For example, the researchers asked people to complete automobile tax forms and varied whether they had to sign prior to or after providing the number of miles driven in the past year. Those signing first reported more miles than those signing last, indicative of less cheating (see for similar findings Mazar et al., 2008). With this research in mind, one could argue that the affirmative answers to ethical statements elicited by the MTQ may not necessarily imply that an individual is currently actively committed to honesty.

Taken together, interventions aimed at reducing symptom over-reporting may prove more effective if they do not only expose individuals to moral reminders but also stimulate a focus on the ethical connotations of the decision at hand and one’s own desire to be an honest person (i.e., increased self-awareness). We aimed to incorporate these ingredients in the design of study 2.

## Study 2: Moral Contracts

Given that the setup employed by Shu et al. (2011) is easily amendable for use in clinical settings, we opted for a variant of this design in study 2. Note that this setup also allowed for a conceptual replication of the Horner et al. (2017) study into the corrective effect of handouts that stress the importance of honest responding. More specifically, we provided treatment-seeking individuals with a moral contract prior to SVT completion and compared their scores with those of individuals who did not receive such a contract. We theorized that presenting participants with a moral contract prior to testing makes them more mindful of the situation-specific relevance of the moral reminders as well as their own desire to be an honest person (i.e., self-awareness). Together, these elements may foster anticipated dissonance that promotes honest symptom reporting.

### Method

#### Participants

Forty-one treatment-seeking individuals (for ADHD complaints) were recruited within the neuropsychology department of PsyQ, an outpatient mental health clinic. Participants were randomly allocated to either the contract (*n* = 21) or no-contract (*n* = 20) condition by means of a coin toss procedure. Given that five participants had missing data on our main outcome measure, we excluded them and ended up with a final sample of *N* = 36 (25 men; *M*_age_ = 32.9 years, SD = 10.9; *n* = 19 in the contract and *n* = 17 in the no-contract condition). The standing ethical committee of the Faculty of Psychology and Neuroscience of Maastricht University as well as the ethical committee of the clinic gave approval for the study.

#### Measures and Procedure

Participants received verbal and written information regarding the study—again without explicit reference to SVTs, hypotheses, or conditions—and were asked to give their consent. The SIMS was our main outcome measure. More specifically, we constructed two half versions of the SIMS to locate potential within-subject changes over time. We aimed for two half versions that contained a balanced number of items of each of the original subscales as to reduce the likelihood that potential effects are actually the result of differences in the representation of symptom domains across time points.^3^ Given that the SIMS (and its subscales) consists out of an odd number of items, it cannot perfectly be divided over two time points. Therefore, we report the proportion of endorsed symptoms (%) rather than total scores. For all participants, half of the SIMS was administered at intake (i.e., baseline; Cronbach’s alpha = .73) and the other half during a session that was specifically scheduled for neuropsychological testing (i.e., posttest; Cronbach’s alpha = .73).

The initial phase was similar for participants regardless of condition. That is, they received general verbal information regarding the importance of performing to one’s best ability during neuropsychological testing (i.e., standard procedure) and subsequently completed the first SIMS. The second session, however, differed between conditions in an important respect: whereas participants in the no-contract condition only received verbal information regarding the importance of sufficient effort and honesty during testing, participants in the contract condition were given the same information in written form and requested to sign as to state their willingness to put forth best effort during testing (see Appendix B for the contract). In addition to the second SIMS, participants completed the Brief Symptom Inventory (BSI; Cronbach’s alpha = .97; De Beurs, 2011; Derogatis, 2000) and the ADHD Rating Scale (ADHD-RS; Cronbach’s alpha = .95; Kooij et al., 2005). The BSI is a widely used instrument that screens for psychological distress in the areas of anxiety, depression, and somatization. By means of 5-point Likert scales (0 = not at all, 4 = always), respondents indicate to what extent they experienced symptoms in the past week. In the present study, we obtained a total score for the BSI by summing across items. The ADHD-RS is a screening tool for ADHD-related symptoms. It is based on ADHD criteria as formulated in the Diagnostic and Statistical Manual of Mental Disorders, fourth edition (DSM-IV; APA, 1994) and consists of 23 items that assess the presence and severity of current (i.e., past 6 months) ADHD symptoms via Likert scales (0 = rarely or never, 3 = very often). Items are summed to obtain a total score.

### Results and Discussion

Table 2 shows the proportion of endorsed symptoms (%) on the SIMS and the mean scores on the BSI and ADHD-RS for the two conditions. To test whether or not signing a contract affects SIMS scores, we conducted a 2 (contract vs. no contract) × 2 (test) analysis of variance (ANOVA) with repeated measures on the second factor.^4^ We did not observe a significant interaction between condition and test (*F* (1, 34) = .16, *p* > .05, *η*_p_^2^ = .00) nor did we obtain a main effect of condition (*F* (1, 34) = .91, *p* > .05, *η*_p_^2^ = .03), although participants in the contract condition obtained slightly higher scores than controls for the SIMS at both time points. A significant main effect was found for test (*F* (1, 34) = 11.08, *p* = .002, *η*_p_^2^ = .25), with SIMS scores decreasing from time 1 to time 2.

**Table 2:** Study 2: mean SIMS endorsement rates (SD) and mean scores (SD) on the BSI and ADHD rating scale per condition

	Condition	
	Contract (*n* = 19)	No contract (*n* = 17)
SIMS 1^a^	17.2 (9.7)	14.3 (11.4)
NI scale	15.8 (15.7)	8.4 (12.4)
AF scale	35.5 (21.4)	28.7 (27.5)
P scale	12.0 (18.6)	7.6 (10.3)
LI scale	9.9 (12.2)	10.3 (10.1)
AM scale	11.3 (11.3)	15.1 (19.9)
SIMS 2^a^	13.9 (10.9)	10.7 (7.7)
NI scale*	11.2 (17.6)	0.7 (3.0)
AF Scale	29.3 (17.5)	28.6 (20.8)
P Scale	7.9 (15.7)	2.2 (4.9)
LI Scale	6.8 (12.0)	5.0 (7.0)
AM Scale	13.2 (12.1)	14.7 (17.3)
BSI^b^	64.2 (44.6)	52.3 (35.8)
ADHD-RS^b^	36.1 (17.4)	34.4 (13.4)

*SIMS* Structured Inventory of Malingered Symptomatology, *BSI* Brief Symptom Inventory, *ADHD-RS* Attention Deficit Hyperactivity Disorder Rating Scale

*p < .05

^a^For both time points, SIMS total and subscale scores are based on half versions of the original SIMS

^b^BSI scores are based on *n* = 17 per condition (two participants in the contract condition had missing data). For the ADHD-RS, there were 16 patients in the no-contract (one patient had missing data) and 17 in the contract condition (two patients had missing data)

A *t* test comparing SIMS scores at time 2 between conditions failed to reach significance (*t* (34) = − 1, *p* > .05, Cohen’s *d* = .34). The corresponding Bayesian factor was 1.21 (i.e., in favor of the null hypothesis). Next, we carried out *t* tests to compare the two conditions with regard to their endorsement rates on the subscales of the SIMS at time 2 (see Table 2). Differences failed to reach significance (all *p*s > .05), except for the neurological impairment (NI) scale: The contract condition scored significantly higher on this scale than the no-contract condition (*t* (19.9) = −2.5, *p* = .02, Cohen’s *d* = .81). However, when applying a Bonferroni correction for multiple testing (*α* = .05/five subscales = .01), this effect disappeared.

Using independent *t* tests, we also examined whether differences between the two conditions emerged regarding BSI and ADHD-RS total scores. Again, no significant differences were found between conditions, with *t* (32) = −.86, *p* > .05, Cohen’s *d* = .29, for BSI, and *t* (31) = −.30, *p* > .05, Cohen’s *d* = .11 for ADHD-RS total scores. Bayesian factor scores were 1.23 and 1.11 for BSI and ADHD-RS scores, respectively (i.e., in support of the null hypothesis).

In sum, study 2 suggests that having patients sign a moral contract is not superior to usual procedures in terms of suppressing over-reporting on the SIMS nor does it result in lower self-reported pathology on the BSI and ADHD-RS. Study 2—like study 1—included individuals seeking treatment for ADHD complaints. It is unknown whether our findings would generalize to other treatment-seeking samples. Furthermore, we relied on non-validated shortened versions of the SIMS. We tried to balance the two halves of the SIMS as much as possible, but one could argue that a more ideal design would have included the complete SIMS at both time points. We refrained from this in the present study because of time restrictions but an additional consideration is that presenting the full SIMS twice might induce a repetition effect.

Aside from these limitations, what could explain why moral reminders—again—did not have impressive effects on symptom over-reporting tendencies? As pointed out before, moral behavior is affected by a drive for consistency, but it also hinges upon a self-regulatory moral balancing system that enables individuals to occasionally permit themselves to engage in undesirable behavior (i.e., moral licensing). This complementary system interprets positive moral primes as an affirmation of moral virtuousness rather than as a cue signaling the importance of committing to honest conduct in the situation at hand. Indeed, researchers have observed that people give themselves credit for having positive intentions even if they do not act upon them and that this allows them to engage in less ethical behavior without facing repercussions to their moral self-concept (e.g., Kruger & Gilovich, 2004). Thus, when moral reminders are phrased positively, they do not always challenge individuals’ self-concept but sometimes rather bolster it and can affect subsequent behavior via moral licensing. As an illustration, Sachdeva, Iliev, and Medin (2009) asked participants to write self-relevant stories containing either positive or negative moral trait words and compared them with controls who received a list of neutral, inanimate words. Those receiving positive primes (e.g., loyal, honest) donated less money to charity, thus showing a licensing effect. Intriguingly, those receiving negative primes donated the most. Authors have proposed that when faced with negative moral primes, individuals need to compensate more strongly because of the more obvious discrepancy that a negatively framed moral self-evaluation poses in relation to their desired moral self-concept (i.e., negative primes induce higher dissonance). To make up for this imbalance, they engage in moral cleansing (see also West & Zhong, 2015).

Although our participants signed a moral contract that should theoretically have alerted them to their desire to respond honestly within the current context (i.e., a consistency effect because of a salient conflict between over-reporting and the desire to be an integer individual), it is possible that participants conceptualized their signing of the contract as confirming their sense of being a morally virtuous individual, which may—paradoxically—have allowed some of them to subsequently license over-reporting. This could explain why we did not obtain the consistency effect reported in previous research, as it would suggest that our participants were not faced with the task of having to repair a threatened moral self-concept. It may thus elucidate why we observed minimal differences between our conditions. With this possibility in mind, we conducted study 3 and took a closer look at whether moral reminders differentially affect feigning depending on their valence (i.e., positive or negative). If so, this may partly explain the null-findings in studies 1 and 2. Furthermore, and contrary to both our initial expectations and the Horner et al. (2017) recommendations, if interventions that rely on moral reminders produce paradoxical effects, it is unadvisable to use them in clinical practice.

## Study 3: Moral Paradox

For study 3, we recruited adults from the general population so that we could use more intricate manipulations to study the impact of differently valenced primes on dissonance and symptom over-reporting. We used the paradigm employed by Sachdeva et al. (2009) to manipulate the valence of our moral reminders and embedded it into a procedure aimed at implicitly motivating participants to over-report symptoms. Our study thus mirrored real-life events where people are tempted to over-report symptoms not because of instructions but because it is somehow beneficial to them (e.g., to obtain a financial incentive). This allowed us to scrutinize moral primes and their underlying forces within a larger sample and under experimentally controlled conditions. We wanted to explore the possibility that if there is any corrective potential of moral reminders for symptom over-reporting it should be most pronounced in individuals presented with negative primes. We reasoned that these primes should result in the highest motivation to repair the moral self-concept (i.e., via moral cleansing; lower symptom over-reporting), whereas positive primes might be readily taken to confirm that one is virtuous and foster moral licensing (i.e., higher symptom over-reporting). To gauge exaggeration, we employed both a version of the SIMS and a measure of underperformance (i.e., the b Test; see below).

### Method

#### Participants

We recruited participants using the SONA recruitment system (i.e., an online recruitment platform through which students from the university can sign up for research), Facebook, flyers, and word-to-mouth advertising. Participation in the study took place via Qualtrics, a web-based research platform that provides participants access to online studies. Originally, we aimed for 156 participants (based on a power calculation). However, 234 individuals entered the study, of which 102 did not complete any measures. Thus, our final sample consisted of 132 adult individuals. Although we aimed for the general population, a median age of 21 suggested that primarily students participated and the majority of participants (83%) were women. Participants were allocated to the positive (*n* = 41), negative (*n* = 38), or neutral (*n* = 53) priming condition.^5^ The standing ethical committee of the Faculty of Psychology and Neuroscience of Maastricht University gave approval for the study.

#### Measures and Procedure

The study was advertised as investigating the links between cognition and psychological well-being. Participants gave consent and were allocated to the positive prime, negative prime, or control condition. In the prime conditions, participants were shown five words encompassing moral traits. In the positive condition, words had a positive connotation (i.e., kind, honest, trustworthy, unselfish, and loyal), whereas in the negative condition, they had a negative connotation (i.e., disloyal, evil, dishonest, selfish, and egoistic). Controls received a list with inanimate words that were unrelated to morality (i.e., chair, computer, stapler, desk, and paper). Participants were led to believe they would have to study these words for later memory recall (i.e., cover story), after which we instructed them to generate self-relevant action sentences for recent events in which the given words applied to them (see Appendix C). Based on previous research, we reasoned that remembering recent self-relevant actions while incorporating these words should prime moral self-concept.

After the prime, participants completed the dissonance affect questionnaire, on which they indicated the degree to which three dissonance-related affect items (i.e., uncomfortable, bothered, and uneasy (Cronbach’s alpha = .48) and three general positive affect items (i.e. filler items: active, inspired, and proud; Cronbach’s alpha = .88) applied to them (i.e., not at all, a little bit, somewhat, very much, and extremely; Harmon-Jones, 2000). This allowed us to measure the psychological discomfort experienced after priming and provided an indication of anticipated cognitive dissonance—which should be most pronounced in the negative prime condition. In line with Harmon-Jones (2000), we calculated an average dissonance score based on the average scores on the dissonance-related affect items.

Next, participants were told the following tasks to be particularly challenging for individuals with symptoms of a learning disability or ADHD, and that if they showed low performance or endorsed symptoms on these tasks, this would make them eligible for an extra compensation of €2.50 (i.e., money-voucher as described in cover story; see also Appendix C). In reality, these tests were two SVTs: the b Test and the brief Dutch version of the SIMS (Cronbach’s alpha = .85) as described by Malcore, Schutte, Van Dyke, and Axelrod (2015).

The b Test (Boone et al., 2000) is a performance-based SVT that requires participants to detect all *b*s among rows and columns of the letters *q*, *d*, and *p*. Although a decreasing letter size over trials makes it seem as if the test becomes more difficult over time, even individuals with severe psychopathology can complete the test successfully. The b Test has been validated in samples of suspected malingerers and participants with various clinical problems (e.g., learning disability, schizophrenia, and moderate to severe head injury). In the present study, we adapted the b Test into a shortened online form. That is, our participants saw five webpages (i.e., as opposed to 15 pages in the original booklet) with rows containing the letters “b,” “q,” “d,” and “p.” Specifically, each page contained 20 *b*s*,* 17 *q*s, 19 *d*s, and 16 *p*s. With each subsequent webpage, the letter font was reduced to create the illusion that the task was getting increasingly difficult. Participants were asked to click on all the *b*s as fast as possible without losing accuracy. We calculated b Test errors by summing up omission errors (overall missing *b*s; range = 0–100) and commission errors (overall endorsement of *d*s*, q*s*,* or *p*s; range = 0–260), respectively. Note that, to our knowledge, the b Test has not previously been administered in online form.

For the SIMS (Malcore et al., 2015), we calculated the total endorsement rate. The abbreviated (i.e., 35 items) SIMS has only four subscales: NI, affective disorder (AF), psychosis (P), and amnestic disorder (AM). Given that not all original SIMS subscales are retained in the short version, we focused on the total score rather than subscale scores. After completing the b Test and brief SIMS, participants underwent an exit interview regarding the purpose of the study, were debriefed, and received monetary compensation (i.e., €7.50 voucher) for their participation.

### Results and Discussion

Table 3 shows mean b Test, SIMS, and dissonance scores for the total sample and per condition. Given that assumptions for parametric tests were violated, we relied on non-parametric testing when assessing differences across conditions. A Kruskal-Wallis *H* test showed that omission errors differed significantly between conditions (*χ* ^2^ (2) = 6.08, *p* = .04, two-tailed) with a mean rank omission error score of 68.96 for the positive, 76.75 for the negative, and 57.25 for the neutral priming condition. Effect size analysis revealed a Cohen’s *d* of .35, suggesting a small to medium effect of priming condition. A Mann-Whitney pairwise comparison test revealed that the negative condition led to significantly more omission errors than the control condition (*p* = .01, two-tailed), but that there was no significant difference between the positive and negative priming condition (*p* > .05, two-tailed) nor between the control and the positive priming condition (*p* > .05, two-tailed). With regard to b Test commission errors, there was no significant difference between conditions as determined by Kruskal-Wallis *H* test (*χ* ^2^ (2) = 1.30, *p* > .05, two-tailed). Similarly, a Kruskal-Wallis *H* test showed no significant differences among conditions regarding SIMS total scores (*χ* ^2^ (2) = .74, *p* > .05, two-tailed).

**Table 3:** Study 3: mean b Test omission and commission errors, SIMS scores, and cognitive dissonance scores per priming condition

	Condition			
	Positive prime (*n* = 41)	Negative prime (*n* = 38)	Controls (*n* = 53)	Total sample (*n* = 132)
b Test omission	5.22 (5.23)	5.36 (3.99)	3.43 (2.87)*	4.54 (4.12)
b Test commission	0.19 (0.67)	0.18 (0.69)	0.07 (0.33)	0.14 (0.56)
SIMS	3.17 (3.64)	3.78 (4.74)	3.79 (4.16)	3.59 (4.17)
Dissonance	0.76 (0.84)	1.28 (0.88)*	0.81 (0.75)	0.93 (0.84)

The values in this table represent participants’ uncorrected scores. Dissonance = bothered, uncomfortable, and uneasy

*SIMS* Structured Inventory of Malingered Symptomatology

*p < .05

To establish cognitive dissonance as a mediator between moral primes and symptom over-reporting, two criteria must be fulfilled: the priming condition must have a statistically significant effect on cognitive dissonance scores and these scores must independently have a statistically significant effect on symptom over-reporting (Baron & Kenny, 1986). A Kruskal-Wallis *H* test showed a significant difference in average dissonance scores between conditions (*χ* ^2^ (2) = 10.07, *p* = .007, two-tailed), with mean rank dissonance scores of 56.99 for the positive priming condition, 82.54 for the negative priming condition, and 62.36 for controls. Effect size analysis revealed a Cohen’s *d* of .50, suggesting a medium effect size. A Mann-Whitney pairwise comparison test revealed that participants in the negative priming condition experienced significantly more dissonance than those in the positive priming condition (*p* < .05 two-tailed) and controls (*p* < .05, two-tailed), whose dissonance scores did not significantly differ from each other (*p* > .05, two-tailed). This suggests that negative primes increased participants’ cognitive dissonance scores.

Next, we investigated the relationship between dissonance scores and feigning for b Test omission errors, b Test commission errors, and SIMS scores separately. Notably, prior to these analyses, we did a log transformation for all three dependent measures to counter normality violations. A simple linear regression using dissonance scores to predict b Test omission errors resulted in a non-significant regression equation (*F* (1, 130) = 1.77, *p* > .05), with an *R* ^2^ of .013. Similarly, we found a non-significant regression for b Test commission errors (*F* (1, 130) = 3.55, *p* > .05), with an *R* ^2^ of .027. This suggests that dissonance did not predict omission nor commission errors. In contrast, dissonance scores significantly predicted SIMS scores (*F* (1, 130) = 11.91, *p* = .001, with an *R* ^2^ of .084, and *R* = .290, explaining 8.4% of variance. Higher dissonance was accompanied with a higher rather than a lower endorsement of bizarre symptoms on the SIMS.

In sum, although negative primes induced higher levels of dissonance when compared with positive and neutral primes (i.e., in line with our expectations), the valence of primes did not have large differential effects on b Test or SIMS scores. Rather, differences were subtle and inconsistent over tests. This observation underscores that moral reminders probably do not lend themselves well for addressing over-reporting in clinical contexts.

## Grand Analysis

To increase the power of our analysis, we collapsed the data of the positive prime conditions vs. the neutral conditions across the three studies (study 1 *N* = 51, study 2 *N* = 36, study 3 *N* = 94) and examined if there was an observable suppressive effect of moral primes on subsequent symptom over-reporting. This resulted in a sample of 181 individuals of whom 97 had been allocated to the neutral and 84 to the prime condition. Our dependent variable was proportion of symptoms endorsed on the SIMS (i.e., the proportion of 75 items in study 1, 38 in study 2, time 2, and 35 in study 3). The mean symptom endorsement rate in the neutral condition was *M* = 13.26% (SD = 13.10), whereas in the prime condition, it was *M* = 12.28% (SD = 11.99). An independent samples *t* test revealed that these rates were not significantly different (*t* (179) = .52, *p* > .05, Cohen’s *d* = −.08). The corresponding Bayes factor is = 1.19. In other words, even with an increased power to detect an effect, our data remain in favor of the null hypothesis: moral primes do not elicit meaningful effects on symptom over-reporting.

## General Discussion

Although presenting people with moral reminders has been found to reduce undesirable behaviors across a range of domains (see Freijy & Kothe, 2013; Mazar et al., 2008; Shu et al., 2011), our findings indicate that such methods are not effective in reducing symptom over-reporting tendencies. We observed a non-significant pattern in the hypothesized direction (i.e., less over-reporting) when exposing patients to moral primes (study 1) but could not replicate the trend observed by Merckelbach and Collaris (2012). Furthermore, no effect—or potentially a marginal backfire effect—occurred when presenting patients with a moral contract (study 2). The Bayesian factor scores were low (< 2), suggesting that there is no firm empirical ground for the idea that moral reminders suppress over-reporting. Furthermore, if they elicit any effect, the findings of study 3 suggest that it is subtle and quite inconsistent in nature. Indeed, notwithstanding the size of the aggregated sample within our grand analysis (*N* = 181), symptom endorsement rates were not significantly different between primed participants and controls. This null finding is in line with what Horner et al. (2017) documented in their study for their total patient sample; there was no effect of the intervention on SVT failure rates.

What may account for the discrepancy between our findings and previous social psychological research on the use of moral reminders to discourage unethical behavior? One possibility is that our manipulations did not elicit sufficiently high levels of anticipated dissonance. As a result, participants may not have felt the need to adjust their subsequent behavior. Indeed, in their pilot study, Merckelbach and Collaris (2012) found that the Mother Teresa prime only modestly succeeded in activating moral categories. Testing an additional number of patients (i.e., study 1), we found no significant correlation between total MTQ and SIMS scores (*r* = −.10, *p* > .05). In study 2, we aimed to increase the salience of moral reminders by accentuating their relevance in the present situation and by adding a component of self-evaluation, yet our findings showed a small (but not significant) trend in the opposite direction. Although the effect size obtained for total SIMS scores at time 2 were small (Cohen’s *d* = .34), the effect for the NI scale was of medium size (Cohen’s *d* = .81), which may suggest that instructions that require individuals more explicitly to commit to honesty might encourage rather than discourage symptom reporting. Indeed, Bargh and Chartrand (2000) noted that in contrast to subtle primes, explicit primes may have less effect, and sometimes even a backfire effect, on subsequent behavior. This may seem an appealing explanation for some of the inconsistencies within our own findings, but it fails to provide sufficient explanation as to why researchers have repeatedly found a positive effect of both subtle primes and more explicit primes (e.g., moral contracts) on behavior in other domains than the one studied in this paper (e.g., Mazar et al., 2008; Shu et al., 2011).

Studies have found that people differ in their sensitivity to moral information. Aquino and Reed (2002), for example, found that moral information has a stronger effect on subsequent behavior in individuals who perceive morality to be of central importance to their identity than in those with a lower moral identity (see also Mulder & Aquino, 2013). In line with such notions, our lab recently found that individuals exhibiting psychopathic traits—and who may thus place less value on morality—are less susceptible to dishonesty-related dissonance than individuals who possess such traits to a lesser extent (Niesten et al., 2015; see also Murray, Wood, & Lilienfeld, 2012). Given that we did not take into account individual difference variables relating to dissonance susceptibility or sensitivity to moral cues, the suppressing effect of moral reminders on over-reporting may have been obscured in our studies. Therefore, we cannot rule out the possibility that moral reminders do, at least in some individuals, discourage symptom over-reporting. Interestingly, Horner et al. (2017) found that stressing the importance of honest responding had a corrective effect on underperformance in individuals who admitted seeking disability benefits but not in those who did not report such benefits (although they might have been present). Although the effect was certainly not large (i.e., Cohen’s *d* = .26), one interpretation is that those who admitted to benefits displayed a higher centrality to internal moral standards and consequently were more susceptible to the corrective effect of the handout compared with those who denied the presence of such benefits (but see below). While individual differences must also have been at play in social psychological studies that found corrective effects after exposure to moral reminders, the large sample sizes of these studies (e.g., Mazar et al., 2008, study 1: *N* = 229, study 2: *N* = 207) may have buffered against the impact that such variability has on the total effect size. Nevertheless, although subtle priming effects might become visible when using larger samples, it is noteworthy that such effects might be too small in magnitude to be of clinical relevance, a point that is underscored by the findings from study 3 and our grand analysis of the data.

In addition to individual difference variables, research suggests that situational factors affect susceptibility to moral cues. This is noteworthy because most studies applied moral reminders in a non-clinical population, whereas we tested their effects in treatment-seeking samples (in studies 1 and 2). Some of our participants may have been actively—and desperately—pursuing long-term benefits that come with receiving a diagnosis (e.g., academic advantages, psychostimulant medication) and these benefits may have had personal significance to them. Indeed, van Egmond and Kummeling (2002) reported that up to 40% of the patients in their sample admitted having a “hidden” agenda containing such motives—and frequently, they had not disclosed these motives to their therapists. In contrast, the desire to obtain benefits was likely less pronounced prior to being presented with the opportunity to cheat among the healthy participants in social psychological research on moral reminders (e.g., Mazar et al., 2008). In tempting situations, acting in self-serving ways seems to happen automatically (Shalvi, Eldar, & Bereby-Meyer, 2012); when incentives become more salient, people’s awareness of moral cues decreases. Using eye-tracking technology, Pittarello, Leib, Gordon-Hecker, and Shalvi (2015) found that when people were given a higher pay off for high dice outcomes in a dice game, they paid less attention to undesirable (i.e., ethical) and more attention to tempting information (i.e., money) than when the payoff depended on accuracy. This lack of attention resulted in a higher occurrence of ethical failures (i.e., more cheating; see also Pittarello, Motro, Rubaltelli, & Pluchino, 2015).

Diminished attention for moral cues is particularly likely in situations high in ambiguity. Thus, ambiguity further blurs the line between right and wrong (Barkan, Ayal, & Ariely, 2015). This is interesting because psychological symptoms can be conceptualized as ambiguous: Symptoms are often subjective in nature and their severity varies over time (Myin-Germeys et al., 2009). Additionally, diagnostic instruments frequently require patients to recall past instances of experiencing symptoms (e.g., the past week) that are likely biased in memory and to indicate the severity of symptoms on rating scales that, because of their format, introduce additional ambiguity (see Slovic & Monahan, 1995). The inherent ambiguity of psychopathology and its assessment—combined with the desire to obtain certain benefits—may nurture peoples’ over-reporting tendencies by allowing them to deny the ethical implications of their act. The additive effect that these variables have on the processing of moral information may thus explain why moral reminders did work in previous social psychological research but had a disappointing effect in our studies. Indeed, whereas previous work has dealt with overt behavior (e.g., cheating to obtain money), over-reporting relates to misrepresenting internal, subjective experiences. Due to the blurred demarcation between what qualifies as genuine and dishonest symptom reporting, individuals can more easily rationalize their deviant reporting in ways that do not require much regard of moral self-concept and simultaneously obscure to themselves any suspicious motives for their behavior. This may explain why in study 3 even negative primes, which should make individuals most invested in exhibiting compensatory behavior (i.e., moral cleansing) to reinstate their moral self-concept, did not result in more accurate responding even though our manipulation closely resembled that used in previous research (e.g., Sachdeva et al., 2009) and was applied within a similar sample.

Both a lack of attention and ambiguity ease the use with which individuals employ self-deceptive strategies to buffer against (anticipated) dissonance. People have a broad repertoire of self-deceptive strategies to choose from at different points in time (see for examples Barkan et al., 2015; Cooper, 2007; Shalvi, Gino, Barkan, & Ayal, 2015); That is, they can dampen the threat that committing an unethical act poses on their self-concept by engaging in pre-violation justifications, by distancing themselves from ethical connotations *during* the violation, or by using post-violation justifications that, for example, allow them to refrain from categorizing the transgression as unethical (Ayal & Gino, 2011). We did not assess justification strategies in our studies. Yet, research into which justification strategies people use when over-reporting symptoms is warranted; Justifications may serve as a malleable mediator that determines the effect of (anticipated) dissonance on behaviors. For example, if individuals fail to categorize over-reporting symptoms as dishonest, reducing ambiguity in testing materials may be essential. Indeed, when people cannot easily justify their unethical behavior, they tend to feel bad (Shalvi et al., 2012), suggesting that discouraging the use of justifications may increase honesty and thus, in the case of feigning, may have a positive effect on the validity of self-reported symptoms. Several authors have also pointed out that justifications allow for more extensive lying (Shu & Gino, 2012; Welsh, Ordóñez, Snyder, & Christian, 2014) and make people less aware of the wrongness of their acts (i.e., ethical fading; Tenbrunsel & Messick, 2004). With such considerations in mind, exploring the effect of even the subtlest forms of (intentional) symptom over-reporting is a goal worth pursuing in future studies, as what may happen over time is that justifications blur the true origin of reported symptoms and eventually facilitate adaptation of the sick role.

Of course, there are likely many factors (e.g., ambiguity and self-justification) that foster and maintain feigning and cause it to escalate into less conscious symptom reporting over time. However, systematic data on candidate factors is largely lacking. To identify these factors, research efforts might concentrate on developing lab paradigms that allow for studying symptom over-reporting and its accompanying cognitive mechanisms in an ecologically valid way (e.g., such as provided in study 3; see for other examples Niesten et al., 2017). Systematic documentation of patient characteristics as well as situational factors that may aggravate—or mitigate—symptom over-reporting tendencies could inform and complement these research endeavors. Together, these lines of study may improve the conceptualization of feigning, which in turn may have important ramifications for how clinicians tackle symptom over-reporting in their patients.

In closing, our studies show that, notwithstanding the large corrective potential that has been ascribed to moral reminders in other fields that dealt with dishonest responding, clinicians should not expect them to have impressive effects in the field of symptom exaggeration. In fact, the Horner et al. (2017, p. 9) conclusion that such a type of intervention could provide “substantial benefit with essentially no cost” seems far too premature: closer inspection of their data shows that the absolute gain in valid SVT scores among patients admitting to disability benefits was quite low (i.e., 6; 16 vs. 22 failures in the no-intervention condition) and the data do not fully preclude the possibility that the observed effect is the result of more sophisticated feigning (Youngjohn et al., 1999). Among patients who did not disclose interest in benefits, the intervention was accompanied by more rather than less *invalid* scores (i.e., 5; non-significant). This pattern of findings does not only suggest that if moral reminders work they may only do so in a subset of individuals who are willing to admit that disability benefits play a role in their symptom reporting but also that they are likely to be an ineffective strategy for discouraging over-reporting among individuals who do not acknowledge such benefits. Indeed, this type of intervention seems to have too unpredictable an effect to confidently implement it as a method to counter symptom over-reporting tendencies. Instead, we recommend researchers to focus on more sophisticated interventions that take into account the complexities surrounding ethical decision-making in patients, particularly in those with a hidden agenda because their alternative motives for seeking treatment may hamper the unbiased processing of moral cues.

## Appendix 1: Mother Teresa Questionnaire

1. I support the notion that people who are suffering from starvation—for instance in Africa—should receive financial support from richer countries.
2. If I was on my way to an important meeting and a passerby would be having a stroke, I would stop to call an ambulance.
3. If I had to choose between a nice evening out with a good friend and visiting a lonely and sick family member, I would choose to visit the family member.
4. I think it is good that victims of highly violent crimes get the chance to explain the consequences of the crime to the perpetrator in court.
5. Even though it’s costs society money, I think that children with disabilities should have the right to good accommodation and educational facilities.
6. I think it’s good when my country organizes national charities to help victims of grave disasters, although I understand that sometimes some of the money will not end up where it is supposed to.
7. If I was a doctor and made a medical error with a patient, I would honestly admit my mistake and to not beat about the bush.
8. I think it’s inappropriate when people throw a button in the donation boxes of volunteers who go from door to door to collect money for the cancer foundation.
9. If an elderly man, on his way to a funeral, would cause a car collision that did not cause any damage, I would certainly not tell his insurance company that I had any damage.
10. I think that people who have saved a child from drowning while risking their own lives deserve a medal of honor.

## Footnotes

1 A copy of the MTQ can be obtained from the first author.

2 The SIMS data were slightly skewed to the right and, based on the Shapiro-Wilk test, violated the normality assumption. However, transforming the data using square root did not result in alternative outcomes. We therefore present the original data.

3 We could not completely counter the issue of unbalanced scale representations because each subscale of the SIMS contains 15 items. However, we tried to minimize it: for the subscales N, P, and AM Scale, seven items were included in SIMS-1 and eight items in SIMS-2 (and vice versa for the AF and LI subscales). Because our two half forms of the SIMS could not be equally divided (time 1, 37 items; time 2, 38 items), we report endorsement rates (%) rather than mean total scores.

4 The assumption of normality was violated for both conditions at time 2, as assessed by the Shapiro-Wilk test. Although a square root transformation partially resolved the violation of normality, main findings did not differ between transformed and non-transformed data. We therefore present the original data.

5 The 102 individuals who did not provide any data were also allocated to the conditions because they had entered the study. This is why we ended up with unbalanced conditions for our final sample.
